# How logical reasoning mediates the relation between lexical quality and reading comprehension

**DOI:** 10.1007/s11145-015-9613-9

**Published:** 2016-02-05

**Authors:** Eliane Segers, Ludo Verhoeven

**Affiliations:** Behavioural Science Institute, Radboud University, Montessorilaan 3, P.O. Box 9104, 6500 HE Nijmegen, The Netherlands

**Keywords:** Logical reasoning, Syllogistic reasoning, Lexical quality, Reading comprehension

## Abstract

The present study aimed to examine the role of logical reasoning in the relation between lexical quality and reading comprehension in 146 fourth grade Dutch children. We assessed their standardized reading comprehension measure, along with their decoding efficiency and vocabulary as measures of lexical quality, syllogistic reasoning as measure of (verbal) logical reasoning, and nonverbal reasoning as a control measure. Syllogistic reasoning was divided into a measure tapping basic, coherence inferencing skill using logical syllogisms, and a measure tapping elaborative inferencing skill using indeterminate syllogisms. Results showed that both types of syllogisms partly mediated the relation between lexical quality and reading comprehension, but also had a unique additional effect on reading comprehension. The indirect effect of lexical quality on reading comprehension via syllogisms was driven by vocabulary knowledge. It is concluded that measures of syllogistic reasoning account for higher-order thinking processes that are needed to make inferences in reading comprehension. The role of lexical quality appears to be pivotal in explaining the variation in reading comprehension both directly and indirectly via syllogistic reasoning.

## Introduction

Reading comprehension encompasses both lower and higher level language skills (Cain, Oakhill, & Bryant, 2004). Lower level skills entail the ability to decode, as well as the quality of the mental lexicon (Perfetti & Hart, 2002). However, to gain a full understanding of a text and accomplish a situation model, the reader has to make inferences. The ability to do so can be seen as a higher level language skill (e.g. Oakhill & Cain, 2012). Inferencing requires the reader to make logical steps in deducing a conclusion based on premises stated in the text. This helps in filling in details that are not explicitly mentioned in the text. The ability to make inferences partly predicts reading comprehension; poor comprehenders have inadequate inferencing skills (Cain & Oakhill, 1999). A distinction can be made between two types of inferences: those that are made with little or no effort during reading, and those that are made after the text has been read and require more effort (Graesser, Singer, & Trabasso, 1994). Less-skilled comprehenders are especially poor in generating the more effortful, elaborative, type of inferences (Bowyer-Crane & Snowling, 2005). The Lexical Quality Hypothesis (Perfetti, 2007; Perfetti & Hart, 2002), mainly focuses on the quality of the mental lexicon in explaining differences between readers. On the one hand, inferencing may be seen as complementary to the quality of the mental lexicon, but there may also be an indirect relation of lexical quality to reading comprehension via inferencing. In the present study, we made an attempt to find out in what way the two different types of inferencing skills mediate the relation between lexical quality and reading comprehension, and to what extent they explain additional variance in reading comprehension.

Lexical quality refers to the pivotal role of word meaning with regard to reading comprehension (Perfetti & Stafura, 2014). Readers need both a large lexicon, as well as high-quality representations of the words within their lexicon. The Lexical Quality Hypothesis (Perfetti, 2007; Perfetti & Hart, 2002) argues that the richness of word representation helps both lower and higher level processes in reading comprehension. In longitudinal studies, it has been found that with progression of grade the role of word decoding as predictor for reading comprehension becomes smaller because of increasing reading fluency, whereas the role of vocabulary becomes larger (Protopapas, Sideridis, Mouzaki, & Simos, 2007; Verhoeven & Van Leeuwe, 2008). Word decoding can be seen as a sine qua non for reading comprehension (Hoover & Gough, 1990). Especially the speed of decoding is relevant with regards to lexical quality, as a high speed of word decoding is an indication of a high quality of phonological and orthographic word knowledge. Perfetti and Stafura (2014) also pointed to the connection of decoding with word meaning, in the sense that in reading comprehension, word meaning has to be activated via the written form of a word. The reader with a good, high-quality, orthographic word knowledge and a broad vocabulary knowledge can read the text at a high speed, and can establish fast word-to-text integration.

In order for word-to-text integration to take place, and thus to comprehend a text, readers have to make inferences, using both prior knowledge and propositional meaning within the sentences of the text at hand (Perfetti & Stafura, 2014). These higher-level processes might be complementary to lexical quality (see also Perfetti, 2007), and partially mediating the relation between lexical quality and reading comprehension (e.g., Cromley & Azevedo, 2007). In their Constructionist Theory, Graesser et al. (1994) described two types of inferences: (1) those that are generated during the course of comprehension to establish both local and global coherence (coherence inferences), and (2) those that are generated after the text has been read (elaborative inferences), for example during a retrieval attempt. This latter type of inferencing is not required for maintaining coherence, but may create a more vivid mental picture of the text. A reader may only make such inferences during reading a text when (s)he has a specific goal and when the inferences are highly predictable. It takes more effort to make these inferences, and exactly this effort is needed to construct meaning and to learn from text (Kintsch & Rawson, 2005). Elaborative inferences are thus often not generated during reading, since they are time-consuming, and their use is highly subject to individual differences (Thurlow & Van den Broek, 1997). Bowyer-Crane and Snowling (2005) showed that questions in a reading comprehension task that require these elaborative types of inferences made the difference between skilled and less-skilled comprehenders (9-year-olds). In a study by Cain, Oakhill, Barnes, and Bryant (2001), 7- to 8-year old skilled comprehenders overall made more inferences but not relatively more elaborative inferences than less skilled comprehenders. And contrary to the expectations, the children made similar amounts of elaborative and coherence inferences. The authors presumed that this might be due to the fact that the children were not yet fluent readers.

In research on thinking and reasoning, the case has been made that there are two separate cognitive systems underlying reasoning (Evans & Stanovich, 2013; Wason & Evans, 1975). The first system processes rapid, automatic, and unconscious, and is shared between humans and animals. The second system is believed to be specifically human. It is slow, conscious, and affords testing of hypotheses. The connection to different types of inferences during reading comprehension is a small next step. Graesser, Wiemer-Hastings, and Wiemer-Hastings (2001) related different types of inferences in reading comprehension to propositional logic and syllogistic reasoning which involves integration of information, drawing of inferences, and consideration of alternative states (Johnson-Laird & Bara, 1984). They suggested that logical reasoning might be a way of capturing the quite varying coherence preserving processes that occur in text comprehension. Inferences like modus ponens are made automatically, and do not take much effort. Indeed, in a masked priming study, Reverberi, Pischedda, Burigo, and Cherubini (2012) evidenced the automaticity of modus ponens. And although modus tollens does not seem to be automatic, this is also a logical type of inferences that is made with little effort, and high accuracy, even in primary school children (see e.g., Haars & Mason, 1986). Modus ponens and modus tollens thus may capture the ability to draw the more easy coherence inferences.

Pseudo-logical inferences cause more problems for participants, but performance increases when the problems are made concrete (Wason, 1968; Evans, 2003). Negation of antecedent and affirmation of consequence are examples of such more difficult, indeterminate, types of syllogisms. Haars and Mason (1986) investigated whether primary school children could solve different types of syllogisms. When a “maybe” answer, instead of a simple “yes” or “no” was required, error rates were higher, but children in the upper primary school grades were able to solve the indeterminate problems. These type of syllogisms may capture the ability to make the more demanding elaborative inferences in reading comprehension, as readers have to come up with various situations before the “maybe” answer can be given. Examples of the different types of syllogisms are presented in Table 1 (based on Schröder, Bödeker, & Edelstein, 2000).

**Table 1:** Different types of syllogisms

Premise	All seal that get a tasty fish are happy	
Modus ponens (affirmation of antecedent)	Seal 1 gets a tasty fish. Is he happy?	Yes
Negation of antecedent	Seal 2 did not get a tasty fish. Is seal 2 happy?	Maybe
Modus tollens (negation of consequent)	Seal 3 is sad. Did seal 3 get a tasty fish?	No
Affirmation of consequence	Seal 4 is happy. Did seal 4 get a tasty fish?	Maybe

The link between syllogistic reasoning and reading comprehension has been made before. For example, two previous studies investigated whether print exposure would positively affect syllogistic reasoning (as a measure of cognition), based on the idea that readers have to make deductions in order to understand a text. The more practice they have (i.e. the more they read), the better they become. Both studies also included reading comprehension measures, but syllogistic reasoning, and not reading comprehension was the dependent variable. Siddiqui, West, and Stanovich (1998) found very modest support for their hypothesis that print exposure fostered syllogistic reasoning, but their results did show positive correlations between syllogistic reasoning and reading comprehension in college students. Osana, Lacroix, Tucker, Idan, and Jabbour (2007) found that exposure to scientific texts that included more logical forms predicted syllogistic reasoning in undergraduate students, and again that logical reasoning was related to reading comprehension.

In the present study, we examined the relation between lexical quality and reading comprehension, and the mediating role of syllogistic reasoning in this relation. Inferencing is a higher-language skill that may be complementary to lexical quality. However, lexical quality may partly encompass the easy, logical type of inferences, as high word knowledge facilitates easy word-to-text integration; theoretically, lexical quality should thus (partly) predict inferencing, and not the other way around. In the present study, we first expected that lexical quality (operationalised as decoding efficiency and vocabulary, cf. Verhoeven, Van Leeuwe, & Vermeer, 2011) would be related to both logical and indeterminate syllogistic reasoning, and that both lexical quality and syllogistic reasoning would be related to reading comprehension. Second, we expected both easy, logical and more difficult, indeterminate syllogisms to predict reading comprehension after controlling for nonverbal reasoning. However, when performing a mediation analyses with a model containing all four variables, and controlling for nonverbal reasoning, we expected direct effects of decoding and vocabulary to reading comprehension. We furthermore expected an indirect effect via indeterminate syllogisms to reading comprehension, while the indirect effect via logical syllogisms was expected to be small or even absent.

## Method

### Participants

Participants were 146 children in 4th grade (74 boys, 72 girls). The children came from seven groups of five different schools in the central-east of the Netherlands. Their average age was 121 months (SD = 5.6 months). The parental educational level was at university (either university or university of applied sciences) level for almost one-third of the group (father 32.2 %, mother 27.4 %), at vocational level for about one-third of the group (father 34.2 %, mother 35.6 %) and below that for the remainder part of the group (father 26.7, mother 30.8 %). The educational level of father and mother was unknown for respectively 6.8 % and 6.2 %. Most children (90.4 %) were monolingual, the others spoke Dutch and Turkish (6.8 %), or Dutch and another language (2.7 %).

### Materials

*Nonverbal reasoning* was assessed with the Raven’s Standard Progressive Matrices (Raven, Raven & Court, 1998). In this task, children are asked to solve five sets of 12 puzzles of which a part is missing. They have to choose out of six items. The puzzles increase in difficulty. Each correctly solved puzzle was scored with one point. The total raw score was used for the present study. Reliability of this test is high, with a reported median of .90.

*Decoding Speed* was assessed with a paper-and-pencil lexical decision task in which children were asked to cross out pseudowords in a list of 120 bisyllabic words (Van Bon, 2007). The words were presented on one page with four columns, which contained 90 nouns, and 30 pseudowords. Children were given 1 min to complete the test. The score is the number of words judged within a minute, minus the number of errors. Reliability is considered good with alpha’s around .80.

*Vocabulary* was assessed with a standardized test in which children were asked to find the synonym of an underlined word in a sentence out of four alternatives (Verhoeven & Vermeer, 1993). A (translated) example item is: “The children are very enthusiastic. (A) intently, (B) funny, (C) absently (D) exuberant”. The paper-and-pencil test consisted of 50 items. The total score comprises the number of correct answers. Reliability is .87 (Verhoeven & Vermeer, 1996).

*Syllogistic reasoning* was measured with two subtests based on a syllogistic reasoning test of Schröeder, Boedeker, and Edelstein (2000). Children read short stories consisting of three sentences. They were instructed as follows: “Below are short stories. At the end of each story, you get a question. Answer each questions with either “yes”, “no” or “maybe”. Only encircle the one answer that you are sure is correct.” The first subtest consisted of 10 items that tapped the more basic reasoning skills, and included easy, logical syllogisms as modus ponens and tollens. The answers to these syllogisms were “yes” or “no”. Another set of 10 items tapped the more complex reasoning skills, and included indeterminate syllogisms that required a “maybe” answer. In all 20 items, answering possibilities were “yes”, “no”’, and “maybe”. The 20 (translated) items can be found in the “Appendix”. An example of a yes/no and a maybe item are: All seals that get a tasty fish are happy. (A) Seal 1 gets a tasty fish. Is seal 1 happy? (answer: yes). (B) Seal 2 did not get a tasty fish. Is seal 2 happy? (answer: maybe). Both subtests turned out reliable, with Cronbach’s alpha being respectively .78 and .72. The data of both subtests were furthermore normally distributed with skewness (−.69 and .40) and kurtosis (−.48 and −.71) within normal ranges.

*Reading comprehension* was assessed by the schools with a standardized Item Response Theory based scale that covers text-based and situation-based comprehension in a range of measures, including summarizing, implicit and explicit questions, and inferring novel word meanings from text (Staphorsius & Krom, 1998). The test consists of three parts, each containing several texts and 25 multiple-choice questions. Each child makes two parts. After the first, general, part, children receive the second part. Based on their score on the first part, this is the more easy or difficult part. The total number of correct answers is transformed to a scale score. The scale was developed by the Dutch National Institute for Measurement in Education (Cito) and is part of a tracking system used by most schools in the Netherlands to monitor children’s abilities during primary school. Reliability and validity are considered “good” according to the Commission on Testing Matters (COTAN) of the Netherlands Institute of Psychologists (NIP).

### Procedure

The schools comprised a convenience sample of schools that worked with the authors on a larger project on science and technology. The first author contacted five schools, that all were willing to participate in exchange for results of the tests. Parents of the children were informed via a letter, and had the opportunity to indicate that they did not want their child to participate. None of them did, and as such, all approached parents gave passive consent for the participation of their child.

Data was assessed in three sessions that took place in the classrooms, with the teacher of the children being present. Five undergraduate students Educational Science were trained, and were each assigned to one of the schools. The students received course credits for their help in data collection. The students explained the procedure of each test in the classroom. The tests were divided over three blocks that were assessed in various orders within the seven classes. Assessment took about 45 min per block. The present data is part of a larger dataset regarding science and technology. Not all children were present at the three occasions of testing, and at points they did not fill in all items in the tests. The data thus contained missing values that were kept as such in the analyses, explaining the slightly varying numbers of participants in the “Results” section.

## Results

### Descriptive statistics

As a preliminary check, we tested whether children scored equally well on the logical syllogisms, the Syllogistic Yes (*M* = 3.41, *SD* = 1.53*)* and No (*M* = 3.33, *SD* = 1.47*)* items. This was indeed the case, *t*(136) = .75, *p* = .46, *d* = .05. Furthermore, the Pearson correlation between the two was high (*r* = .65, *p* < .001). Following our hypotheses and the results of these analyses, the scores were therefore collapsed for further analyses.

Table 2 presents the descriptive statistics. Whereas children on average scored 67.4 % correct of the Logical Syllogisms, this score dropped to 37.4 % for the Indeterminate Syllogisms. This was a statistically significant difference, *t*(135) = 8.30, *p* < .01, *d* = 1.17. Regarding the Logical Syllogisms, 35 % of the children score 9 or 10 points, whereas only 3.6 % reached such a high score for the Indeterminate Syllogisms. However, 26.4 % of the children scored 6 or more points in this latter category.

**Table 2:** Descriptive statistics of nonverbal reasoning, decoding speed, vocabulary, syllogistic reasoning, and reading comprehension

Variables	n	M	SD	Min	Max
Nonverbal reasoning	145	40.12	7.54	11	55
Decoding speed	144	60.19	17.89	21	110
Vocabulary	141	32.38	7.87	8	47
Logical syllogisms	137	6.74	2.72	0	10
Indeterminate syllogisms	136	3.74	2.46	0	10
Reading comprehension	142	31.73	15.39	0	85

In Table 3, the correlations between the variables under study are presented. As expected, Nonverbal Reasoning, Decoding, Vocabulary, and both types of Syllogistic Reasoning were related to Reading Comprehension, and to each other, with only two exceptions. The Indeterminate Syllogisms had a correlation approaching zero with Decoding, whereas this was not the case for the Logical Syllogisms. On the other hand, the Indeterminate Syllogisms correlated significantly with Nonverbal Reasoning, while the Logical Syllogisms did not. Furthermore, especially the negative correlation between the two syllogistic reasoning tests is noticeable. Some children with a very high score on the Indeterminate Syllogism items scored a bit lower on the Logical Syllogism items. On the other hand, there were some children that hardly ever came up with a Maybe answer, while having a high score at the Yes/No items.

**Table 3:** Pearson correlations between predictor measures and criterion measure (n = 135–142)

	1	2	3	4	5	6
1. Nonverbal reasoning	–					
2. Decoding speed	−.02	–				
3. Vocabulary	.31***	.28**	–			
4. Logical syllogisms	.14	.25**	.37***	–		
5. Indeterminate syllogisms	.29**	.00	.22*	−.34***	–	
6. Reading comprehension	.34***	.38***	.68***	.36***	.28**	–

* *p* < .05; ** *p* < .01; *** *p* < .001

### The role of syllogistic reasoning in reading comprehension

We first performed a hierarchical linear regression analysis to investigate the unique effects of Syllogistic Reasoning on Reading Comprehension, without taking lexical quality into account. In Step 1, Nonverbal Reasoning was entered as a control variable. In Step 2, the two syllogistic reasoning variables were entered: Logical Syllogisms and Indeterminate Syllogisms. The results are presented in Table 4. The final model explained 31 % of the variance, which is a moderate to large effect. Both reasoning scales significantly explained variance in Reading Comprehension, after controlling for Nonverbal Reasoning.

**Table 4:** Results of the stepwise hierarchical regression analysis in predicting reading comprehension (n = 133)

	*∆R* ^*2*^	B	SE (B)	Β
Step 1	.10**			
Nonverbal reasoning		.66	.18	.32**
Step 2	.22**			
Nonverbal reasoning		.30	.16	.14^+^
Logical syllogisms		2.73	.45	.48**
Indeterminate syllogisms		2.55	.51	.41**
Total $R_{adj}^{2}$	.31**			

^+^ *p* < .10; * *p* < .05; ** *p* < .01; *** *p* < .001

### Direct and indirect effects of lexical quality on reading comprehension

To combine all variables under investigation for our final hypothesis, we used the Process add-on in SPSS (Hayes, 2013), and performed a mediation analysis. The two lexical quality measures, Decoding Speed and Vocabulary were the independent variables, the two syllogistic measures (Logical Syllogisms and Indeterminate Syllogisms) were the mediators, and Reading Comprehension was the dependent variable, with Nonverbal Reasoning as covariate. Because of the two independent variables, the model was run twice, each with one of the independent variables as covariable, to be able to estimate the effects. Bootstrapping was set at 5000 cycles, as recommended by Hayes (2013). In mediation models, the total effect c of an independent variable on the dependent variable, is the addition of the direct effect c’ and the indirect effect ab. The indirect effect ab is the product of the effect of the independent variable on the mediator (a) and the effect of the mediator on the dependent variable (b). An indirect effect ab may be significant, even when a or b in itself are not (Hayes, 2013).

**Fig. 1:**
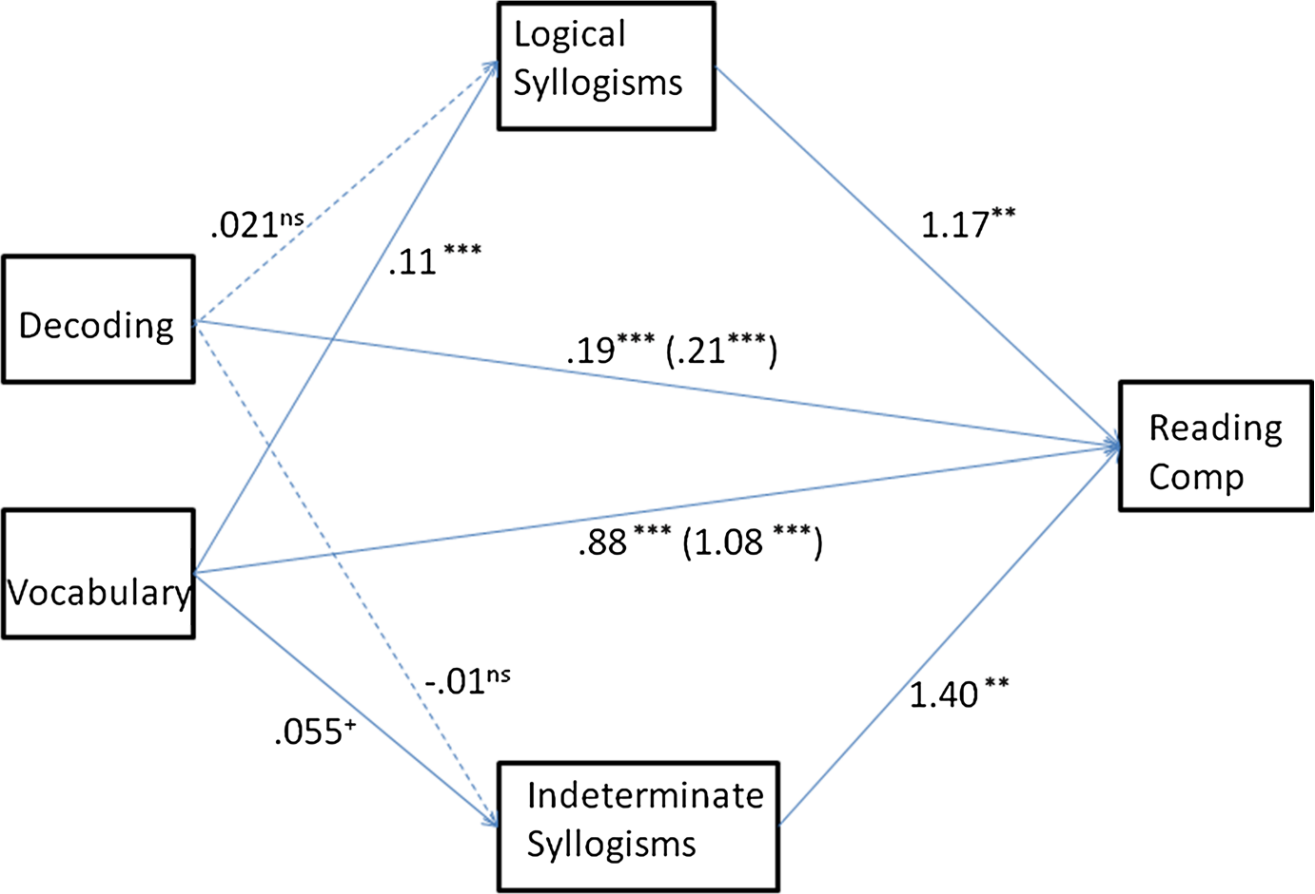
Model for predicting reading comprehension via lexical quality through syllogistic reasoning, while controlling for nonverbal intelligence. Unstandardized coefficients are reported. Between *brackets* are total effects (c), outside the brackets the direct effects (c′). *Note* *p < .05; **p < .01; ***p < .001

Figure 1 depicts the final model, with the unstandardized coefficients. The total R^2^ of the model was .56 (*p* < .001). It shows how Lexical Quality impacts Reading Comprehension, with strong direct effects of both Decoding and Vocabulary on Reading Comprehension. The previously found effects of Syllogisms remained significant in this model.

The total indirect effect of Decoding on Reading Comprehension via both aspects of Syllogistic Reasoning was not significant, as the 95 % confidence interval contained zero (ab = .013, 95 % CI = [−.016–.05]). However, the total indirect effect of Vocabulary on Reading Comprehension via both aspects of Syllogistic Reasoning was significant (ab = .20, CI = [.09–.37]). When we break down this latter effect into its components, the indirect effect ab of Vocabulary on Reading Comprehension via Logical Syllogisms was .12 (CI = [.04–.27]) and the indirect effect of Vocabulary on Reading Comprehension via Indeterminate Syllogisms was .08, (CI = [.01–.20]).

## Discussion and conclusion

The main aim of the present study was to investigate in what way lexical quality, syllogistic reasoning and reading comprehension were related, and whether lexical quality incorporates aspects of inferencing to predict reading comprehension. Inferencing was operationalized in the present study via two types of syllogistic reasoning: easy, logical syllogisms (requiring a yes or a no answer) and more difficult, indeterminate syllogisms (requiring a maybe answer via elaborative inferencing). Both types of syllogisms had a unique additional effect on reading comprehension, on top of lexical quality, but its effects were also indirect, via lexical quality, and more specifically via the breadth of the lexicon. Lexical quality had strong direct effects on reading comprehension, also when syllogistic reasoning was taken into account.

Our first expectation entailed the relations between aspects of lexical quality (decoding efficiency and vocabulary), nonverbal reasoning, syllogistic reasoning, and reading comprehension. As expected, all variables related to each other, but nonverbal reasoning was not associated with easy, logical syllogistic reasoning. This is consistent with the idea that less effort is needed for these types of syllogisms. Furthermore, decoding efficiency was not associated with indeterminate syllogistic reasoning, probably because decoding efficiency is a reading speed measure and not a reflection of a higher-order thinking process. More puzzling is the negative correlation between the two types of reasoning. Some children who were very good in logical syllogisms, scored very low at the indeterminate syllogisms. Perhaps these children have a strategy to hardly ever answer “maybe”, whereas we could speculate that those children that do very well in the indeterminate but poor in the logical syllogisms, tend to overthink the problem, and so are prone to answer “maybe”. Haars and Mason (1986) also showed the difficulty children may have in selecting the “maybe” answer, even though they have full understanding of both premises. The authors suggested that these children stop analyzing the problem prematurely, whenever a possible correct answer is encountered. The overall picture, however, is in line with the dual-processing distinction advocated by Evans (2003) and Evans and Stanovich (2013) suggesting that there are two systems underlying reasoning: one autonomous, automatic system, and one evolutionary newer system that allows hypothetical thinking and abstract reasoning. The term “automatic” should be taken with caution though, as although there is evidence that modus ponens is automatic, this is not the case for modus tollens (Reverberi, et al., 2012).

The second hypothesis entailed the associations of both components of reasoning with reading comprehension, after controlling for nonverbal reasoning. As expected, both were related to reading comprehension, in line with results from Siddiqui et al. (1998), and Osana et al. (2007). When lexical quality aspects (decoding and vocabulary) were taken into account, these effects remained, and indirect effects from lexical quality via syllogistic reasoning on reading comprehension were established. Probably because there was no time constraint in the assessment, especially vocabulary drove the indirect effect, and not so much the decoding efficiency measure. In this respect, it should also be noted that word decoding efficiency in a more transparent language (Dutch in the present study) is less salient as predictor of reading comprehension in the upper grades of primary school (e.g., Verhoeven, et al., 2011).

It appeared that lexical quality does not fully entail the easy, straightforward, inferencing processes, as we had expected. The ability to solve both types of syllogisms made a unique contribution in explaining variation in reading comprehension on top of lexical quality, partly confirming our second hypothesis. The top-down skill is thus not contained in the bottom-up skill, and even the more easy, logic syllogisms add to the prediction of reading comprehension.

Monti and Osherson (2012) posed that deductive reasoning is not necessarily linguistic, but that this possibly only holds for the more easy syllogisms. They claimed that the role of language is most salient in the initial coding of verbal information. In fMRI research, Kuperberg, Lakshmanan, Caplan, and Holcomb (2006) showed that a large bilateral network is activated when processing connected sentences. The discussion is ongoing, and it is difficult to draw a conclusion in this respect based on our results. Following Monti and Osherson, one could, however, argue, that the ability to solve syllogisms is a form of nonverbal intelligence. As a case in point, Shikishima, et al. (2011) suggested a strong association between syllogistic reasoning ability and general intelligence (g). We controlled for this by taking a nonverbal measure of intelligence (nonverbal reasoning) as covariate in our design.

There are some limitations that should be acknowledged at this point. First, we did not incorporate a measure of working memory, while this has an important relation to reasoning (Evans & Stanovich, 2013). In future research, adding this measure will help to more fully understand the associations between lexical quality, reasoning, and reading comprehension. Second, different measures of reading comprehension could be assessed for a more fine-grained of reading comprehension abilities. In the present study, we used a composite measure that could not be disentangled into its components. In future research, it would be interesting to find out whether the logic syllogisms are more associated with forming a text-based model, and the indeterminate syllogisms with forming a situation model. Finally, it should be acknowledged that the present study was a first attempt to connect syllogistic reasoning ability to reading comprehension, in order to get one step closer in understanding individual differences in reading comprehension. This study was the first to combine these two measures in developing readers in primary school. When further studying these relations, it is recommended to also incorporate other, more refined, measures of logical reasoning, and by taking up a longitudinal approach. This may provide more insight in the possible reciprocal relations between the different measures.

Based on the results of the present study, it can be concluded that measures of syllogistic reasoning account for higher-order thinking processes that are needed to make inferences in reading comprehension. However, the role of lexical quality appears to be pivotal in explaining the variation in reading comprehension both directly and indirectly via syllogistic reasoning.

## Appendix

See Table 5.

**Table 5:** Syllogistic reasoning test

	All seal that get a tasty fish are happy	
1	Seal 1 gets a tasty fish. Is he happy?	Yes
2	Seal 2 did not get a tasty fish. Is seal 2 happy?	Maybe
3	Seal 3 is sad. Did seal 3 get a tasty fish?	No
4	Seal 4 is happy. Did seal 4 get a tasty fish?	Maybe
	All children in Hanna’s class like the apples from the fruit stall	
5	Niels likes the apples from the fruit stall. Is Niels in Hanna’s class?	Maybe
6	Renee is in Hanna’s class. Does Renee like the apples from the fruit stall?	Yes
7	Daniel is not in Hanna’s class. Does Daniel like the apples from the fruit stall	Maybe
8	Floor does not like the apples from the fruit stall. Is Floor in Hanna’s class?	No
	All children who are bored during gym class, like to row a boat	
9	Anna is bored during gym class. Does Anna like to row a boat?	Yes
10	Stella likes to row a boat. Is Stella bored during gym class	Maybe
11	Paul does not like to row a boat. Is Paul bored during gym class?	No
12	Peter is not bored during gym class. Does Peter like to row a boat?	Maybe
	There is a observatory near Daniel’s house. He went there a few times after school. When Daniel goes to the observatory, he always takes his bike	
13	Daniel did not go to the observatory yesterday. Did he ride his bicycle yesterday?	Maybe
14	Daniel did not ride his bicycle the day before yesterday. Did he go to the observatory the day before yesterday?	No
15	Daniel rode his bicycle today. Did he go to the observatory today?	Maybe
16	Daniel went to the observatory last week. Did he go by bike?	Yes
	If there is an earthquake in Pisa, the leaning tower of Pisa will fall down	
17	There is an earthquake in Pisa. Will the leaning tower fall down?	Yes
18	There is no earthquake in Pisa. Will the learning tower fall down?	Maybe
19	Imagine the leaning tower of Pisa falls down. Is there an earthquake then in Pisa?	Maybe
20	What if the learning tower does not fall down. Is there an earthquake in Pisa?	No
